# Structural Insights into a Bifunctional Peptide Methionine Sulfoxide Reductase MsrA/B Fusion Protein from *Helicobacter pylori*

**DOI:** 10.3390/antiox10030389

**Published:** 2021-03-05

**Authors:** Sulhee Kim, Kitaik Lee, Sun-Ha Park, Geun-Hee Kwak, Min Seok Kim, Hwa-Young Kim, Kwang Yeon Hwang

**Affiliations:** 1 Department of Biotechnology, Korea University, Seoul 02841, Korea; sulhee@korea.ac.kr (S.K.); kitaeque@gmail.com (K.L.); psh3810@naver.com (S.-H.P.); yhalstjr2@naver.com (M.S.K.); 2 Department of Biochemistry and Molecular Biology, Yeungnam University College of Medicine, Daegu 42415, Korea; waiii@hanmail.net

**Keywords:** MsrAB, fusion protein, linker region, catalytic efficiency

## Abstract

Methionine sulfoxide reductase (Msr) is a family of enzymes that reduces oxidized methionine and plays an important role in the survival of bacteria under oxidative stress conditions. MsrA and MsrB exist in a fusion protein form (MsrAB) in some pathogenic bacteria, such as *Helicobacter pylori* (*Hp*), *Streptococcus pneumoniae*, and *Treponema denticola*. To understand the fused form instead of the separated enzyme at the molecular level, we determined the crystal structure of *Hp*MsrAB^C44S/C318S^ at 2.2 Å, which showed that a linker region (*Hpiloop*, 193–205) between two domains interacted with each *Hp*MsrA or *Hp*MsrB domain via three salt bridges (E193-K107, D197-R103, and K200-D339). Two acetate molecules in the active site pocket showed an *sp*^2^ planar electron density map in the crystal structure, which interacted with the conserved residues in fusion MsrABs from the pathogen. Biochemical and kinetic analyses revealed that *Hpiloop* is required to increase the catalytic efficiency of *Hp*MsrAB. Two salt bridge mutants (D193A and E199A) were located at the entrance or tailgate of *Hpiloop*. Therefore, the linker region of the MsrAB fusion enzyme plays a key role in the structural stability and catalytic efficiency and provides a better understanding of why MsrAB exists in a fused form.

## 1. Introduction

The sensitivity of amino acid residues to oxidation is diverse [1]. Among all amino acids, methionine has a very high tendency to oxidize, and when it is present in high concentrations on the surface of a specific protein it can be oxidized without affecting the function of the protein [1]. For certain proteins, methionine oxidation results in their inactivation or activation [2,3,4]. Methionine sulfoxide reductase (Msr) is an antioxidant enzyme that reduces oxidized methionine. Msr plays an essential role in the survival of bacteria under oxidative stress conditions, as shown in deletion mutants [5,6,7]. Methionine oxidation produces diastereomeric compounds, L-methionine S-sulfoxide and L-methionine R-sulfoxide. Two different classes of Msr, named MsrA and MsrB, show distinct preferences for each diastereomer. MsrA is stereospecific for reducing L-methionine S-sulfoxide, whereas MsrB is stereospecific for the reduction of the R diastereomer [1,5,6,8,9]. The absence of MsrA or MsrB causes increased sensitivity to hydrogen peroxide, implying the importance of reducing Met-(R, S)-O [1,5,6,10,11]. MsrA and MsrB exist as separate enzymes in most organisms; however, more than 46 bacterial species contain the fused form of MsrA and MsrB [12,13,14]. The fused form has two domain sequences, MsrAB (MsrA–MsrB) and MsrBA (MsrB–MsrA) [12]. Currently, it is unclear why some bacteria, especially pathogenic bacteria, such as *Helicobacter pylori* (*Hp*), *Streptococcus pneumoniae*, and *Treponema denticola*, possess MsrAB as a fusion protein instead of individual enzymes [15,16]. The catalytic activities of the MsrAB fusion protein are higher than those of the individual enzymes, MsrA and MsrB [15,16,17,18]. Many structures from each domain have been reported [18,19,20,21,22,23,24]. Two crystal structures of the MsrAB fusion protein have been determined to date [15,16]. In both crystal structures, MsrA and MsrB are connected by a linker region (named the *iloop*) composed of a short peptide that plays a role in the catalytic efficiency or structural stability of the fusion protein; however, the exact function or reason behind the formation of the fusion protein has not been determined to date [15,16]. Various proteins from the human gastric pathogen *H. pylori* are linked to Msr enzymes. Many of these proteins, such as GroEL and recombinase, are more methionine-rich than other bacterial proteins, and are oxidized under oxidative stress and rescued by the activity of Msr enzymes [25]. Thus, Msr proteins repair oxidative damage to methionine residues via the oxidation/reduction cycle, serve as scavengers of reactive oxygen species, and protect cells from more widespread oxidative damage [26,27]. Here, we determined the crystal structure of the fusion MsrA–MsrB protein from *H. pylori* (*Hp*MsrAB) at a resolution of 2.2 Å. In addition, biochemical and kinetic analyses of *Hp*MsrAB, its single domain forms (*Hp*MsrA and *Hp*MsrB), various catalytic mutants, and *iloop* mutants were undertaken. We compared the structure of *Hp*MsrAB with other fused proteins and examined its specific role based on kinetic analyses. These results support the idea that the *iloop* of the MsrAB fusion enzyme plays a key role in the structural stability and catalytic efficiency and provides a better understanding of why MsrAB exists in a fused form and the specific role of the *iloop* in fused proteins from pathogens.

## 2. Materials and Methods

### 2.1. Cloning, Expression, and Purification

The *MsrAB* gene was amplified by polymerase chain reaction (PCR) using *H. pylori* strain 26,695 genomic DNA as a template. The forward and reverse oligonucleotide primers of *Hp*MsrAB (amino acid residues 24–359) were 5′-GGAATTC CATATG gaaaacatgggatctcaacaccaaa-3′ and 5′-CCG CTCGAG tta atgcgactttttatcattgatgtatttt-3′. The underlined bases represent the NdeI and XhoI sites, respectively. The PCR products were digested with NdeI and XhoI and ligated into the pET-28a vector (Novagen, Darmstadt, Germany). Site-directed mutagenesis was performed using the recombinant plasmid as a template (E193A, D197A, E193AD197A, E339A, and Y343F). Sequences were confirmed using commercial DNA sequencing (Bionics Co., Ltd., Seoul, Korea). The confirmed recombinant plasmid DNA and mutants were introduced into the *Escherichia coli* BL-21 Star (DE3) strain (Invitrogen) to measure the expression. Cells were cultured in LB medium, and protein expression was induced with 1.0 mM IPTG at 18 °C at an optical density of 0.6 at 600 nm. After induction, the cells were harvested by centrifugation at 2700× *g* for 30 min at 4 °C and disrupted by sonication in buffer A (20 mM Tris-HCl pH 8.0, 150 mM NaCl, and 3 mM MgCl_2_) with 1 mM phenylmethylsulfonyl fluoride. The crude cell extract was centrifuged at 17,400× *g* for 60 min at 4 °C and applied to a HisTrap HP 5 mL column (GE Healthcare, Chicago, IL, USA). The column was washed with buffer A containing 40 mM imidazole, and the protein was eluted by a linear gradient with buffer A and 40–500 mM imidazole. The eluted protein was concentrated using an Amicon Ultra-15 (molecular weight cutoff 10,000; Merck Millipore, Darmstadt, Germany), and loaded onto a Superdex 75 HiLoad 16/60 column (GE Healthcare) equilibrated with buffer B (20 mM Tris-HCl pH 8.0, 0.1 M NaCl, and 3 mM MgCl_2_). The protein bands were detected by staining with Coomassie Brilliant Blue R. The purified protein was concentrated to a final concentration of approximately 50 mg/mL, determined using Bradford’s method with bovine serum albumin as a standard [28].

### 2.2. Crystallization and Data Collection

The sitting drop vapor diffusion method using various screening kits from Hampton Research (Aliso Viejo, CA, USA) (Crystal Screen, Index, SaltRx, PEG/Ion, PEGRx, Crystal Screen Cryo, and Crystal Screen Lite) and Qiagen (Venlo, The Netherlands) (Classic suites I and II) was used for the initial crystallization screening. Optimization of crystallization was performed using the conventional vapor diffusion method with a 24-well VDX plate (Hampton Research) at 293 K. Hanging drops were established by mixing 1 µL of each protein solution concentrated at approximately 50 mg/mL equilibrated with 500 µL of the reservoir solution. *Hp*MsrAB^C44S/C318S^ (24–359) (100 µM) was incubated with 200 µM of dabsyl-Met-O for 60 min at 4 °C, and the protein was concentrated to approximately 50 mg/mL. Finally, the appropriate crystals of *Hp*MsrAB^C44S/C318S^ (24–359) with Met-(R,S)-O for X-ray diffraction data were obtained in 0.1 M sodium acetate (pH 4.6) and 2.0 M sodium formate. X-ray diffraction data were collected on beamline 11C (Micro-MX) at the Pohang Accelerator Light Source (Pohang, Korea). The wavelength of the synchrotron X-ray was 1000 Å, and the maximum resolution was 2.2 Å. The collected images were indexed, integrated, and scaled using the HKL-2000 program [29].

### 2.3. Kinetic Analysis of MsrAB with an Enzymatic Assay

The dithiothreitol (DTT)-dependent assay using dabsyl-Met-O as a substrate [30] was performed using purified proteins dialyzed into an appropriate buffer containing 50 mM sodium phosphate (pH 7.5) and 50 mM NaCl. The reaction mixture contained 50 mM sodium phosphate (pH 7.5), 50 mM NaCl, 200 µM dabsyl-Met-S-O (for MsrA activity) or dabsyl-Met-*R*-O (for MsrB activity), 0.26–16 µM Msr proteins, and 20 mM DTT [31,32]. The reaction was performed at 37 °C for 0.5 h, and then the product, dabsyl-Met, was evaluated using high-performance liquid chromatography as described previously [10,31]. The kinetic parameters (*K*_m_ and *k*_cat_) were determined by non-linear regression using GraphPad Prism 5 software (San Diego, CA, USA).

### 2.4. Structure Determination and Refinement

The initial model of *Hp*MsrAB^C44S/C318S^ was solved by molecular replacement with MOLREP [33] using a search model (PDB entry: 5FA9 [15]) in the CCP4 suite [34]. Model building was performed iteratively using the COOT [35] and AutoBuild module [36] in PHENIX [37]. Final refinement was accomplished using the PHENIX program. The final model was validated using MOLPROBITY [38] and had R values of *Hp*MsrAB^C44S/C318S^ with an *R*_free_ of 21.4% and an *R*_work_ of 18.8% at 2.2 Å. The data collection and structure refinement statistics of *Hp*MsrAB^C44S/C318S^ are shown in Table 1. All structural figures were produced using PyMOL Molecular Graphics System (Version 2.0 SchrÖdinger, LLC, NY, USA).

### 2.5. Inflection Temperature (T_i_) Measurement

Prior to T*_i_* measurement, wild-type (WT) and various *Hp*MsrAB mutants were diluted to a concentration of 1 mg/mL for Tycho NT.6 experiments. The samples were then loaded as duplicates into Tycho NT.6 capillaries (Cat# TY-C001; NanoTemper Technologies; Munich, Germany). T*_i_* measurements were taken using a Tycho NT.6 (NanoTemper Technologies) and calculated by plotting the first derivative of the 350/330 nm ratio against temperature.

### 2.6. Size-Exclusion Chromatography with Multiangle Light Scattering (SEC-MALS)

SEC-MALS experiments were performed using a fast protein liquid chromatography system (GE Healthcare) connected to a Wyatt MiniDAWN TREOS MALS instrument (Santa Barbara, CA, USA) and a Wyatt Optilab rEX differential refractometer. A Superdex 200 10/300 GL (17-5175-01; GE Healthcare) gel filtration column preequilibrated with 20 mM Tris-HCl (pH 8.0), 100 mM NaCl, and 2 mM DTT was normalized using bovine serum albumin. Individual proteins (*Hp*MsrAB WT, *Hp*MsrA domain, *Hp*MsrB domain, and various mutants, including *Hp*MsrAB^C44S/C318S^, *Hp*MsrAB^D197A^, *Hp*MsrAB^E193A^, *Hp*MsrAB^E339A^, and *Hp*MsrAB^Y343F^) were prepared separately by the methods described earlier and were injected (1–2 mg/mL, 0.25 mL) at a flow rate of 0.8 mL/min. The final purity of the proteins was checked by SDS-gel electrophoresis (Figure S5). Data were analyzed using the Zimm model for static light scattering data fitting and represented using an EASI graph with a UV peak in the ASTRA V software (Wyatt).

## 3. Results and Discussion

### 3.1. Overall Structures of HpMsrAB^C44S/C318S^

**Figure 1:**
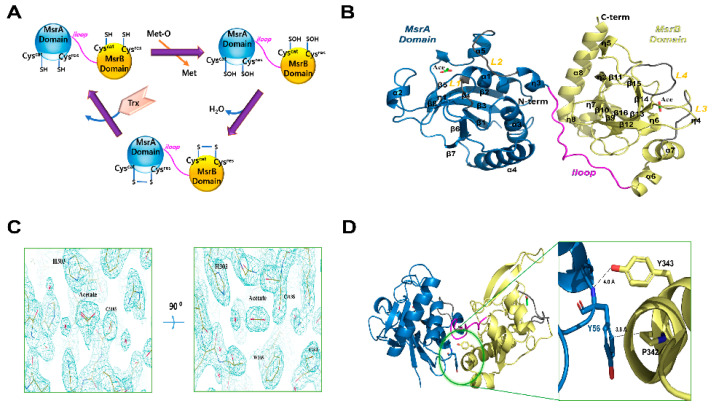
Crystal structure of *Hp*MsrAB. (**A**) Cartoon model of the general catalyzed chemical reactions of MsrAB (**B**) Ribbon diagram of the overall structure of *Hp*MsrAB^C44S/C318S^. The N-terminal domain, MsrA domain (*Hp*MsrA, 34−192) and the C-terminal domain, MsrB domain (*Hp*MsrB, 206−357) are colored sky blue and pale yellow, respectively. The linker region connecting *Hp*MsrA and *Hp*MsrB (residues 193−205), the *iloop*, is colored magenta. Loops (L1, 42−45 and L2, 181−187) of the *Hp*MsrA domain and loops (L3, 224−229 and L4, 259−266) of the *Hp*MsrB domain are colored gray. Helices (α1–α8), β-strands (β1–β16), and 3_10_-helices (η1–η8) are labeled. Two acetate molecules are represented as stick models colored green. (**C**) 2Fo-Fc electron density map (1.2 sigma cutoff) around the acetate molecule in the crystal structure. (**D**) Close-up view of a possible hydrophobic interaction (<4 Å) between Y56 of the *Hp*MsrA domain and P342 of the *Hp*MsrB domain in the crystal structure. The dotted line indicates the distance between the OH of Y343 and the amide N atom of Y56 (~4.0 A), which could not form hydrogen bonds.

**Figure 2:**
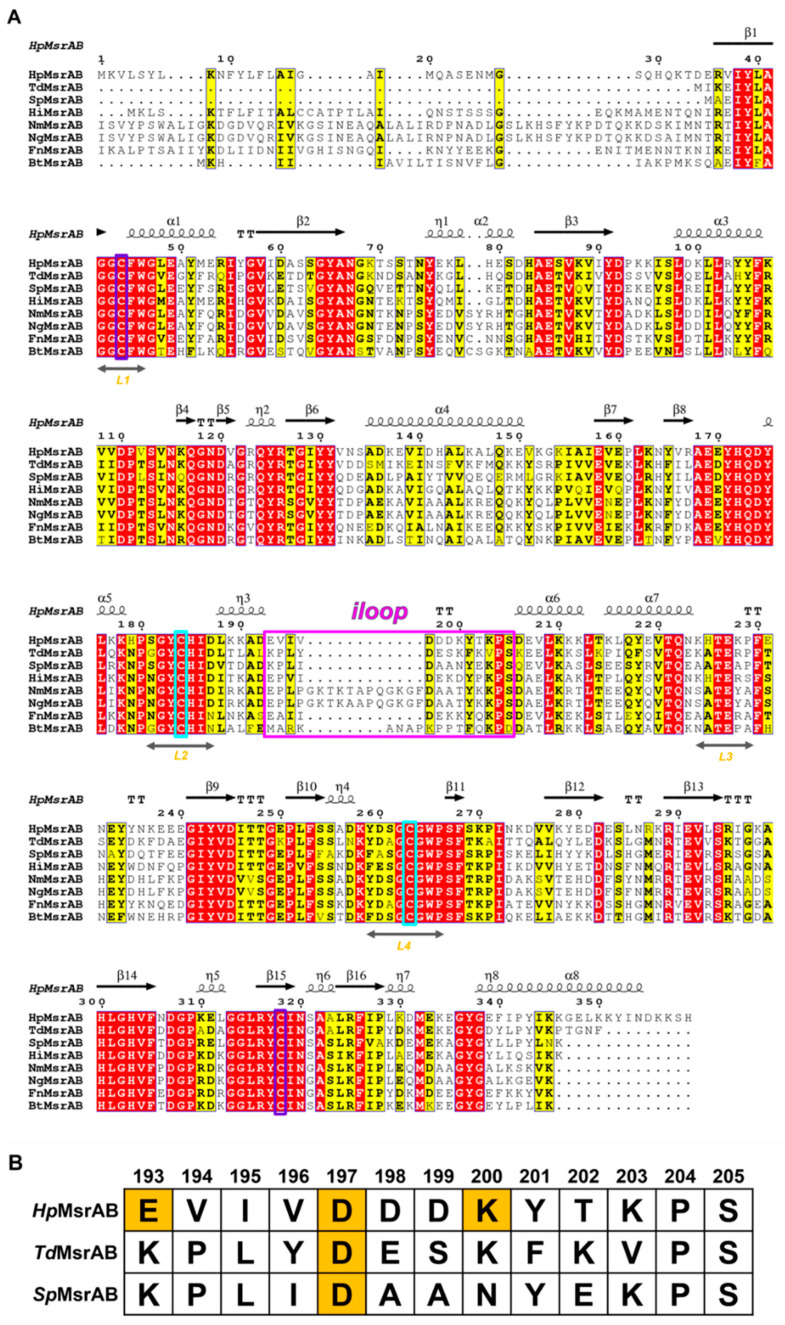
Multiple sequence alignment of *Helicobacter pylori* methionine sulfoxide reductase AB (*Hp*MsrAB). (**A**) Multiple sequence alignment of MsrAB homologs in *Helicobacter pylori* (*Hp*, Swiss-Prot entry O25011), *Treponema denticola* (*Td*, Swiss-Prot entry Q73PT7), *Streptococcus pneumoniae* (*Sp*, Swiss-Prot entry P0A3Q9), *Haemophilus influenzae* (*Hi*, Swiss-Prot entry P45213), *Neisseria meningitidis* (*Nm*, Swiss-Prot entry Q9JWM8), *Neisseria gonorrheae* (*Ng*, Swiss-Prot entry P14930), *Fusobacterium nucleatum* (*Fn*, Swiss-Prot entry Q8R5 × 2), and *Bacteroides thetaiotaomicron* (*Bt*, Swiss-Prot entry Q8A4U8). Catalytic and resolving Cys residues are shown in purple and cyan, respectively. The completely conserved and similar group amino acids are represented by red and yellow letters, respectively. *Hpiloop*, the linker region, is displayed in a magenta box. (**B**) Comparison of *iloops* between *Hp*MsrAB, *Td*MsrAB, and *Sp*MsrAB. Partial sequence alignment of the *iloops* in *Hp*MsrAB, *Td*MsrAB, and *Sp*MsrAB. The residues associated with the salt bridge interactions in *Hpiloops* between MsrA and MsrB domains are shown in orange boxes.

**Figure 3:**
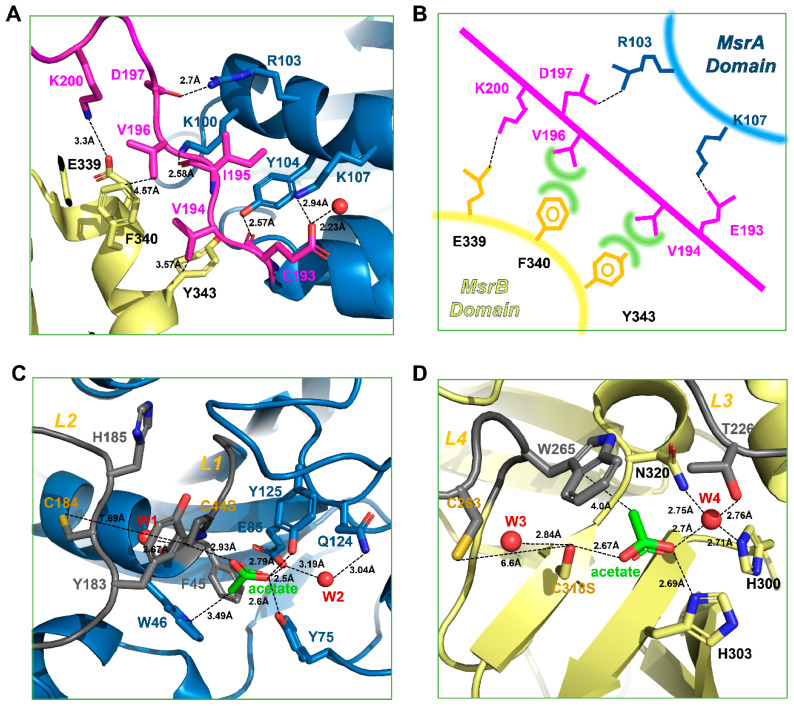
Binding interface and active sites of *Hp*MsrA and *Hp*MsrB. (**A**) Zoomed view of the binding interface between *Hp*MsrA and *Hp*MsrB. The amino acids involved in the interaction are displayed as stick models. The residues (E193, D197, and K200) in the *iloops* are associated with the salt bridge interactions and the residues (E193, V194, and I195) in the *iloops* are associated with the hydrogen bonds or hydrophobic interactions. (**B**) Illustration of salt bridges and hydrophobic interactions. Dashed black lines represent salt bridges, and green curves represent hydrophobic interactions. (**C**) Zoomed view of the active site of the *Hp*MsrA domain. Residues that constitute the active site are displayed as stick models. Two loops involved in forming the active site, L1 (residues 42–45) and L2 (residues 181–187), are colored gray. The acetate molecule is displayed as a stick model colored green. The water molecules are displayed as spherical models colored red. (**D**) Zoomed view of the active site of the *Hp*MsrB domain. Residues that constitute the active site are displayed as stick models. Two loops associated in forming the active site, L3 (residues 224–229) and L4 (residues 259–266), are colored gray.

Initially, we crystallized the catalytic site-specific *Hp*MsrAB^C44S^, *Hp*MsrAB^C318S^, and *Hp*MsrAB^C44S/C318S^ mutants with dabsyl-Met-(R,S)-O, which is a substrate analog compound (Figure 1A). However, a crystal grew in the *Hp*MsrAB^C44S/C318S^ mutant without the substrate. The crystal structure of *Hp*MsrAB^C44S/C318S^ was solved at 2.2 Å and belonged to the P1 space group (Table 1). There were two protomers (MolA and MolB) in the asymmetric unit (Figure S1). We could not identify the electron density map of any substrate in the crystal structure, despite using co-crystallization with dabsyl-Met-(R,S)-O. The purified *Hp*MsrAB^C44S/C318S^ was monomeric in the solution based on SEC and SEC-MALS (Figure S2). Each monomer of *Hp*MsrAB^C44S/C318S^ had two catalytic domains, *Hp*MsrA (34–192, residue number) and *Hp*MsrB (206–357), which were linked by the linker region, *iloop* (193–205) (Figure 1B). The overall fold was similar to the two fusion MsrAB structures (PDB entry: 3e0m and 5fa9) [15,16] (Figure 2A). Each domain, *Hp*MsrA and *Hp*MsrB, was similar to the formerly reported MsrAs and MsrBs, respectively, except for the C-terminal long α-helix (α8) of *Hp*MsrB (Figure 1A and Figure S3). The *Hp*MsrA domain consisted of a central antiparallel β-sheet containing strands β1–β8 surrounded by four α-helices (α1–α4) on the side, one small α-helix (α5), and two 3_10_-helices (η1, η2) (Figure 1B). The *Hp*MsrB domain consisted of two parallel β-sheets that contained three β-strands (β13, β9, and β15), three other strands (β10, β14, and β16), two α-helix bundles (α7 with α6 and long C-terminal α-helix α8) lining both sides of the β-sheets, and three small α-helices (α9–α11) (Figure 1B). The root-mean-square deviations (rmsd) of *Hp*MsrA for *Sp*MsrA (sequence identity 56.6%) and *Td*MsrA (sequence identity 58.2%) were 0.734 Å over 145 Cα atoms and 0.697 Å over 158 Cα atoms, respectively. *Hp*MsrB generated rmsd values of 0.822 Å (141 Cα) and 0.419 Å (141 Cα) for *Sp*MsrB (sequence identity 62.4%) and *Td*MsrB (sequence identity 61.7%), respectively (Figure S3). The *iloop* of *Hp*MsrAB consisted of 13 residues (193–205) and was incorporated into the flank of *Hp*MsrB (Figure 1B). The two domains of *Hp*MsrAB, *Hp*MsrA and *Hp*MsrB interacted with each other via hydrophobic interactions (Figure 1D). There were three salt bridges and several hydrogen bonding interactions in the *iloop* (Figure 3A,B). The first salt bridge was formed between Oδ1 of D197 in the *iloop* and NH1 of R103 in helix α3 of *Hp*MsrA (~2.7 Å). The second salt bridge was formed between Oε1 of E193 in the *iloop* and Nζ of K107 of *Hp*MsrA (~2.94Å). The third salt bridge was formed between Nζ of K200 of the *iloop* and Oδ1 of E339 of *Hp*MsrB (~3.30Å). In addition, two hydrogen bonds were formed between the *iloop* and *Hp*MsrA: one between the carbonyl oxygen atom of I195 in the *iloop* and the Nζ of K100 in *Hp*MsrA (~2.58 Å), and the other between the carbonyl oxygen atom of E193 in the *iloop* and the OH of Y104 in *Hp*MsrA (~2.57 Å) (Figure 3A). Among the three salt bridges, D197 was completely conserved in the fusion of MsrABs from the pathogen (Figure 2A,B). Therefore, these salt bridges played a role in the structural stability and functional catalytic efficiency of the fusion protein MsrAB from the pathogen.

### 3.2. Active Sites of HpMsrAB^C44S/C318S^

The known active sites include highly conserved catalytic cysteine (C44S for *Hp*MsrA and C318S for *Hp*MsrB) and resolving cysteine (C184 for *Hp*MsrA and C263 for *Hp*MsrB) in *Hp*MsrAB (Figure 2A and Figure 3C,D). Many conserved residues were found near the cysteine active site, including -^42^GGCFWG^47^- for MsrA and -^313^GGLRYCIN^320^- for MsrB, which face each other, and histidine residues (H185 for MsrA and H303 for MsrB) that coordinate the oxygen atom of L-methionine sulfoxide [15,34]. These residues within the active site region of *Hp*MsrAB were well conserved across species (Figure 2A). Even though we co-crystallized an *Hp*MsrAB mutant (C44S/C318S) protein with the substrate analog compound, dabsyl-Met-(R,S)-O, no analog compounds were found in the active sites of *Hp*MsrA or *Hp*MsrB. Based on several crystallographic analyses of Msrs in different states (substrate-bound, oxidized, or reduced), our structure showed a reduced-state structure in the catalytic steps [21]. C44S of the *Hp*MsrA domain was positioned in the loop (L1) between strands β1 and α1, and the active site region was formed with two loops (L1, residues 42–45 and L2, residues 181–187) and strand β3 around the C44S residue (Figure 3C). The resolving C184S position was located at L2 and positioned ~7.7 Å from the catalytic C44S. This structure showed that the reduced form of *Hp*MsrA and the oxygen atom of C44S were formed with well-ordered water molecules (~2.67 Å). Y183 and H185, which are well-conserved residues in this region, were expected to participate in the configuration of the active site (Figure 3C). F45 and W46 formed a hydrophobic pocket, and E85 and Y125 contributed to the formation of the active site (Figure 3C). The active site of *Hp*MsrB was positioned in antiparallel β-strands β14 and β15, and two loops (L3, residues 224–229 and L4, residues 259–266) (Figure 3D). The distance between C318S situated on strands β15 and C263 (resolving cysteine) on L4 was ~6.6 Å (Figure 3D). We found two acetate molecules in the active site pocket, which was used as the crystallization buffer (0.1 M sodium acetate) (Figure 1B,C and Figure 3C,D). The two acetate molecules showed an sp^2^ planar electron density map in the crystal structure (Figure 1C). In the active site of the *Hp*MsrA domain, the carboxyl group of the acetate ion interacted with Oδ1 of E85 (2.50 Å), OH of Y75 (2.60 Å), OH of Y125 (2.79 Å), and Oδ of S44 (2.93 Å). The methyl group of the acetate ion interacted with W46 (3.49 Å). Two well-ordered water molecules in the active pocket were found near the active residues. The hydrogen bonding of water (W1) formed with the Oδ1 of C44S (2.67 Å), which is an active cysteine residue. Another water molecule (W2) interacted with Oδ1 of E85 (3.19Å) and Nδ2 of Q124 (3.04Å) (Figure 3C). The binding interaction of the acetate ion of the *Hp*MsrB domain was similar to that of the *Hp*MsrA domain. The interaction residues were as follows: the carboxyl group of the acetate ion interacted with Nε of H303 (2.69 Å), one water molecule (W4, 2.70 Å), and Oδ of C318S (2.67 Å). The methyl group of the acetate ion interacted with W265 (4.0 Å). Two well-ordered water molecules in the active pocket were found near the active residues. The hydrogen bonding of water (W3) formed with Oδ of C318S (2.84 Å), which is an active cysteine residue. The other water molecule (W4) interacted with Oγ of T226 (2.76 Å), Nδ of N320 (2.75 Å), and Nδ of H300 (2.71 Å) (Figure 3D). Overall, the interaction residues of acetate ions were well conserved in the pathogens (Figure 2A). A comparison of the structural locations of the active site residues of *Hp*MsrAB showed that its active site structure was comparable to that of other reduced structures of MsrABs (Figure S3). Therefore, both the active sites of *Hp*MsrAB were reduced.

### 3.3. Biochemical and Kinetic Analysis of HpMsrAB

**Figure 4:**
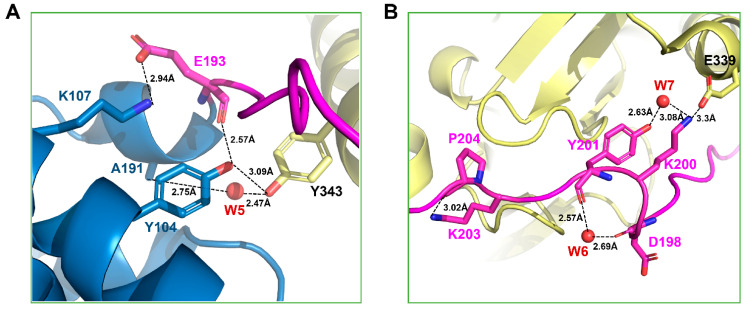
Interactions around the Y343 and E339 residues. (**A**) Magnified view of the interaction residue around Y343. The residues involved in the interaction are represented as stick models. The water molecule is represented as a spherical model colored red and (**B**) Close-up view of the interaction residue around E339.

**Figure 5:**
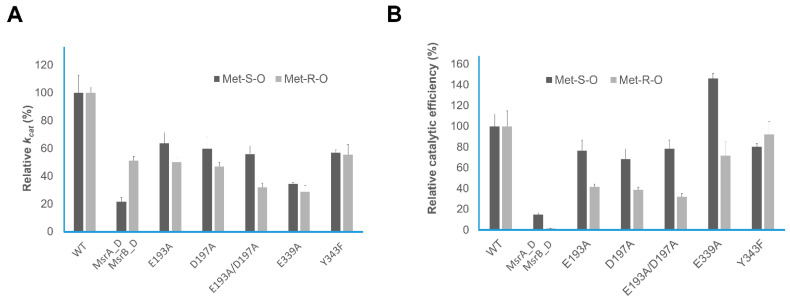
Relative kinetic values. (**A**) Relative *k*_cat_ values of *Hp*MsrAB, *Hp*MsrA, *Hp*MsrB, and *Hp*MsrAB mutants and (**B**) Relative catalytic efficiency (*k*_cat_/*K*_m_) of *Hp*MsrAB, *Hp*MsrA, *Hp*MsrB, and *Hp*MsrAB mutants.

The linker region (*iloop*) linking the two domains (*Hp*MsrA and *Hp*MsrB domain) might play a role in controlling the catalytic efficiency of *Hp*MsrAB, similar to the other fusion MsrAB proteins (*Sp*MsrAB [15] and *Td*MsrAB [16]). The *iloop* might stabilize the spots of each domain by networking with domains via hydrophobic interactions or several hydrogen bonds, as demonstrated in the *Sp*MsrAB and *Td*MsrAB structures [15,16]. The *iloop* of *Hp*MsrAB (*Hpiloop*) was composed of 13 residues (193–205), some of which participated in interactions (salt bridges, hydrogen bonds, or hydrophobic interactions) with *Hp*MsrA and *Hp*MsrB (Figure 3A). *Hpiloop* did not contain any helix structures such as the *iloop* of *Td*MsrAB (*Tdiloop*), whereas the *iloop* of *Sp*MsrAB (*Spiloop*) included helix structures. There was one salt bridge interaction between K200 of *Hpiloop* and D339 of *Hp*MsrB (3.30 Å); however, there were two salt bridges between *Hpiloop* and *Hp*MsrA, namely D197-R103 (2.70 Å) and E193-K107 (2.94 Å). Moreover, there were hydrophobic interactions, such as V196-F340 (~4.6 Å) and V194-Y343 (~3.6 Å), between *Hpiloop* and *Hp*MsrA (Figure 3A,B). These interactions between *Hpiloop* and each *Hp*MsrA or *Hp*MsrB might help the two domains maintain their secondary structures. Therefore, the *iloop* may contribute to the structural stability of each domain in *Hp*MsrAB, similar to other fusion MsrABs. To examine whether the *Hp*MsrAB fusion protein can alter catalytic activity, we performed biochemical assays of several *iloop* mutants of *Hp*MsrAB (Table 2). We prepared several mutants, and blocking the salt bridges between *Hpiloop* and *Hp*MsrB had a significant effect on the enzyme activity of *Hp*MsrAB. We also mutated the other interaction residues, E339A and Y343F, which are located on the C-terminal helix. E339 interacted with K200-E339 via a salt bridge, and Y343 interacted with the carbonyl groups of A191, E193, and water molecules (W5) via a hydrogen bond (Figure 4A,B). We measured the thermal stability of all the proteins to check for stable protein folding (Figure S4). The *k*_cat_ values of *Hp*MsrAB^E193A^ and *Hp*MsrAB^D197A^ for Met-R-O were 2.0- and 2.13-fold lower than that of WT *Hp*MsrAB, respectively. Moreover, the *k*_cat_ value for Met-S-O was 1.57- and 1.65-fold lower than that of the WT, respectively. In the double mutant form of *Hp*MsrAB^E193A/D197A^, the *k*_cat_ values for Met-R-O and Met-S-O decreased by 3.13- and 1.79-fold, respectively, compared to that of the WT (Figure 5A). The *k*_cat_ values of *Hp*MsrAB^E339A^ and *Hp*MsrAB^Y343F^ for Met-R-O were 3.50- and 1.81-fold lower than that of WT *Hp*MsrAB, respectively (Figure 5A). Additionally, the *k*_cat_ values of *Hp*MsrAB^E339A^ and *Hp*MsrAB^Y343F^ for Met-S-O were 2.91- and 1.62-fold lower than thay of the WT, respectively. Small differences in *K*_m_ values were observed for Met-S-O and Met-R-O in the mutants *Hp*MsrAB^E193A^, *Hp*MsrAB^D197A^, and *Hp*MsrAB^E193A/D197A^ (Table 2). However, in *Hp*MsrAB^E339A^ and *Hp*MsrAB^Y343F^, the *K*_m_ value for Met-S-O was 4.25-fold higher than that of the WT *Hp*MsrAB, whereas it was not considerably altered in Met-R-O. Overall, compared with the WT, the catalytic efficiency (*k*_cat_*/K*_m_) of *Hp*MsrAB^E193A/D197A^ was 3.13-fold lower for Met-R-O and 1.28-fold lower for Met-S-O (Figure 5B, Table 2). Compared to the WT, the catalytic efficiency of each domain, *Hp*MsrA or *Hp*MsrB, was 6.82-fold lower for Met-S-O and 67.1-fold lower for Met-R-O (Figure 5B). Overall, substrate binding of the *Hp*MsrB domain could be influenced by the disturbance of the interface between *Hpiloop* and *Hp*MsrA. These biochemical analyses imply that the interaction between *Hpiloop* and *Hp*MsrA plays a role in *Hp*MsrB catalytic activity and that this interaction also influences the catalytic activity of MsrA. In summary, the overall fold or active site configurations of the *Hp*MsrA and *Hp*MsrB domains might be considerably disturbed as they are divided from the fusion *Hp*MsrAB form, suggesting that structural factors influence the catalytic efficiency of *Hp*MsrAB. For *Hp*MsrAB, the catalytic efficiency of the *Hp*MsrB domain was more affected than that of *Hp*MsrA, resulting in an interaction between the *iloop* and *Hp*MsrA. By abolishing these interactions, such as with E193A and D197A, the fusion protein *Hp*MsrAB might not fold stably.

### 3.4. Structural Comparison with Other Fusion MsrAB Proteins

To understand the structural differences between the fusion MsrAB proteins from pathogenic bacteria, *Hp*MsrAB structures were compared with those of *Td*MsrAB and *Sp*MsrAB. The *Hp*MsrAB shared ~51.4% and ~52.9% sequence identity with *Td*MsrAB and *Sp*MsrAB (Figure 2A) and rmsds were ~2.81 and ~1.66 Å for 324 Cα (Figure S3), respectively. When the structures of the MsrA domains of the fusion MsrAB from the three species were superimposed, the MsrB domain orientation was relatively different (Figure S3), which resulted in the flexibility or different hydrogen bonding positions of the *Hpiloop*. For *Neisseria gonorrheae* (*Ng*) PilB, there were reported not full fusion *Ng*MsrAB structure but the domain structures of *Ng*MsrA or *Ng*MsrB [19,39]. Thus, we also superimposed the MsrA or MsrB domain structures of PilB (*Ng*MsrA:PDB1H30 or *Ng*MsrB:PDB1L1D) [19,39] (Figure S3). The rmsds of *Ng*MsrA for *Hp*MsrA and *Ng*MsrB for *Hp*MsrB were 3.88 Å (151 Cα) and 0.779 Å (144 Cα) atoms, respectively. The overall fold of *Ng*MsrB was more similar to that of *Ng*MsrA. The overall structures of the MsrA and MsrB domains from the three species (*H. pylori*, *S. pneumoniae*, and *T. denticola*) were highly similar (Figure S3), and the active site residues were completely conserved (Figure 2A). E193, D197, and K200, which are involved in salt bridge interactions on the *iloop* of *Hp*MsrAB, were affected by the catalytic efficiencies of *Hp*MsrAB; however, these residues were not strictly conserved in fusion MsrABs from the pathogen without the D197 position (Figure 2B). In contrast, no significant interaction sites between *Hp*MsrA or *Hp*MsrB domains were found in *Hp*MsrAB, similar to the *Sp*MsrAB structure. Only one hydrophobic interaction site (Y58-P342) was found in *Hp*MsrAB (~3.8 Å) (Figure 1D). For *Td*MsrAB, they were found in three interacting sites between *Td*MsrA and *Td*MsrB [15,16]. No remarkable structural changes in the active residues, such as C44S, C184, C318S, and C263, were observed between the fusion MsrABs from the three pathogens. Therefore, the interaction of the *iloop* of fusion MsrAB played a key role in the catalytic efficiency or structural stability, and the flexibility of the *iloop* was an important factor depending on the catalytic efficiency of each domain. For *Hp*MsrAB, the flexibility of the *iloop* was controlled via three salt bridges.

## 4. Conclusions

We determined the crystal structure of *Hp*MsrAB^C44S/C318S^ at a resolution of 2.2 Å. The results showed that the *iloop* interacted with *Hp*MsrB via three salt bridges, namely E193-K107, D197-R103, and K200-D339. When the structures of the MsrA domains of the three species (*H. pylori*, *S. pneumoniae*, and *T. denticola*) were superimposed, the orientation of the MsrB domain was relatively different (Figure S3), resulting in the flexibility or different hydrogen bonding positions in the *Hpiloop*. In addition, we found that the linker region (*Hpiloop*) connecting *Hp*MsrA and *Hp*MsrB was mandatory for the higher catalytic efficiency of *Hp*MsrAB based on biochemical and kinetic analyses. We mutated salt bridge mutants (E193A and D197A) located at the entrance or tailgate of the *Hpiloop.* These salt bridges or hydrogen bonding in the *iloop* region play a role in the structural stability and higher functional catalytic efficiency of the fusion protein MsrAB from the pathogen.

## Figures and Tables

**Table 1:** Crystallographic statistics of *Hp*MsrAB^C44SC318S^.

	*Hp*MsrAB^C44SC318S^
**Data collection**	
Wavelength (Å)	1.00000
Resolution (Å)	30–2.2 (2.24–2.20) ^a^
Space group	*P1*
Unit cell parameter	a = 52.125, b = 72.505, c = 82.230 α = 93.16°, β = 93.80°, γ = 111.08°
Observed/unique reflections	158,134/53,424
Redundancy	3.1 (2.3)
Completeness (%)	94.1 (86.6)
*I*/*σ*	29.7 (5.32)
*R*_merge_ (%) ^b^	6.3 (17.9)
**Refinement**	
Resolution (Å)	30.00–2.2
*R*_work_/*R*_free_ (%) ^c^	18.8/21.4
Average *B*-factor (Å^2^)	39.42
Root-mean-square-deviations	
Bond length (Å)	0.006
Bond angle (°)	0.759
Ramachandran favored (%)	97.7
Ramachandran outliers (%)	0.0
PDB entry	5FA9

^a^ Values in parentheses represent the highest resolution shell. ^b^ *R_merge_* = ∑*_hkl_*∑*_i_*|*I_hkli_* − ‹*I_hkli_*›|/∑*_hkl_*∑*_i_*‹*I_hkli_*›. ^c^ *R_cryst_* = ∑*_hkl_*||*F_o_*| − |*F_c_*||/∑|*F_o_*|.

**Table 2:** Kinetic analyses of *H. pyroli* MsrAB, HpMsrA, HpMsrB domain, and its mutants.

Form	Substrate	*K*_m_ (mM)	*k*_cat_ (min^−1^)	*k*_cat_/*K*_m_ (mM^−1^ min^−1^)
WT	Met-*S*-O	0.17 ± 0.06	10.2 ± 1.3	60 ± 7
	Met-*R*-O	0.05 ± 0.02	9.4 ± 0.4	188 ± 8
MsrA_D	Met-*S*-O	0.25 ± 0.09	2.2 ± 0.3	8.8 ± 1.2
MsrB_D	Met-*R*-O	1.7 ± 0.1	4.8 ± 0.3	2.8 ± 0.2
E193A	Met-*S*-O	0.14 ± 0.06	6.5 ± 0.8	46 ± 6
	Met-*R*-O	0.06 ± 0.02	4.7 ± 0.3	78 ± 5
D197A	Met-*S*-O	0.15 ± 0.04	6.1 ± 0.9	41 ± 6
	Met-*R*-O	0.06 ± 0.02	4.4 ± 0.3	73 ± 5
E193A/D197A	Met-*S*-O	0.12 ± 0.05	5.7 ± 0.6	47 ± 5
	Met-*R*-O	0.05 ± 0.02	3.0 ± 0.3	60 ± 6
E339A	Met-*S*-O	0.04 ± 0.01	3.5 ± 0.1	88 ± 3
	Met-*R*-O	0.02 ± 0.01	2.7 ± 0.4	135 ± 25
Y343F	Met-*S*-O	0.12 ± 0.02	5.8 ± 0.2	48 ± 2
	Met-*R*-O	0.03 ± 0.02	5.2 ± 0.7	173 ± 23

WT, wild-type MsrAB form; MsrA_D, MsrA domain form; MsrB_D, MsrB domain form.

## Data Availability

The structure factor and coordinate file have been deposited in the Protein Data Bank (www.rcsb.org, accessed on 11 March 2021) under accession code 7E43.

## Supplementary Materials

The following are available online at https://www.mdpi.com/2076-3921/10/3/389/s1, Figure S1. Overall structure of HpMsrAB in an asymmetric unit, Figure S2. Measurement of molecular masses by SEC-MALS, Figure S3. Comparison of *iloops* between HpMsrAB and SpMsrAB and superimposition of HpMsrAB and SpMsrAB by aligning the MsrA domains, Figure S4. Inflection temperature (Ti) measurement. T*_i_* of *Hp*MsrAB WT and mutants, Figure S5. Image of SDS-gel electrophoresis of WT and mutant HpMsrAB.
