# Supplementary material for: Structural Insights into a Bifunctional Peptide Methionine Sulfoxide Reductase MsrA/B Fusion Protein from Helicobacter pylori
Source: Antioxidants (Basel). 2021 Mar 5;10(3):389. doi: 10.3390/antiox10030389 (PMC8000184; doi:10.3390/antiox10030389)
Supplement: Supplementary file 1 [file antioxidants-10-00389-s001.pdf]

Figure S1

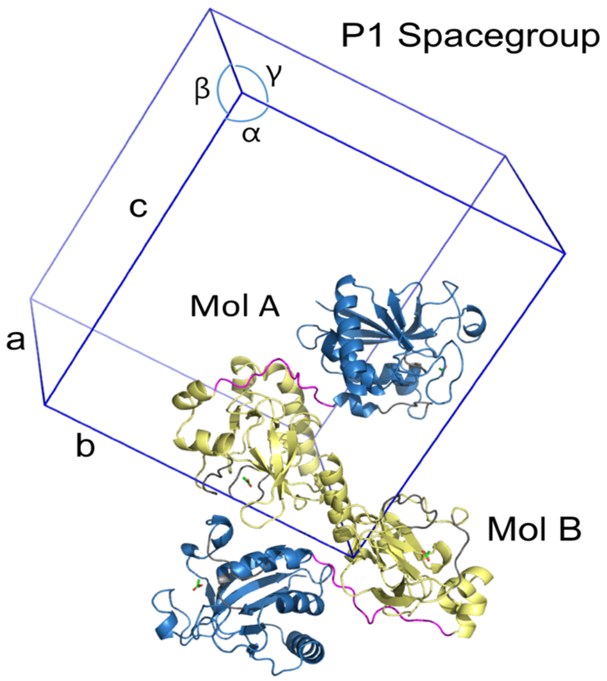

Figure S1: Overall structure of HpMsrAB in an asymmetric unit. The crystal belongs to the P1 spacegroup and two molecules (Mol A and B) in the asymmetric unit. The detailed unit cell parameter is displayed in Table 1.

Figure S2

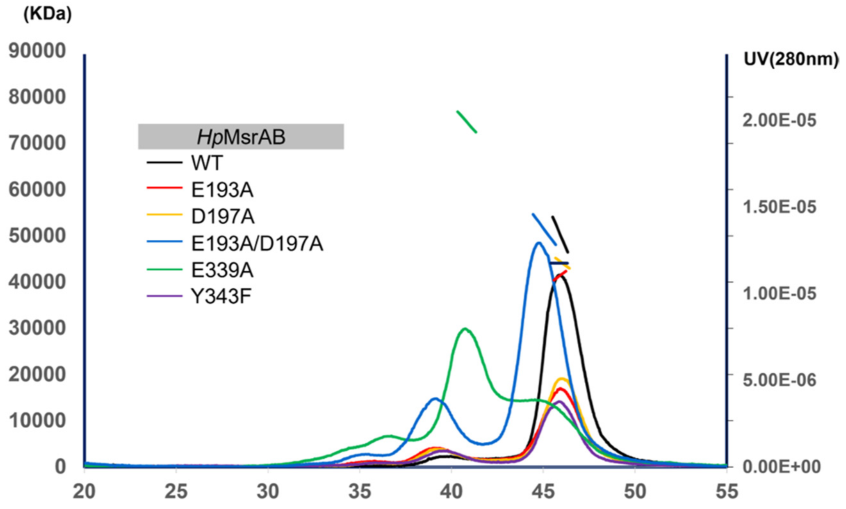

Figure S2: Measurement of molecular masses by SEC-MALS. Exact molecular masses of HpMsrAB wild-type (WT) and mutants were measured by SEC-MALS. Pooling areas for measurement are shown in violet box. Black, red, orange, blue, green, and purple profiles represent WT, E193A, D197A, E193A/E197A, Y343F, and E339A, respectively, and the corresponding horizontal lines represent the measured data obtained by MALS. Each species is indicated by an arrow with experimental (MALS) and theoretically calculated (Calc) molar mass values shown in parentheses (MALS/Calc).

Figure S3

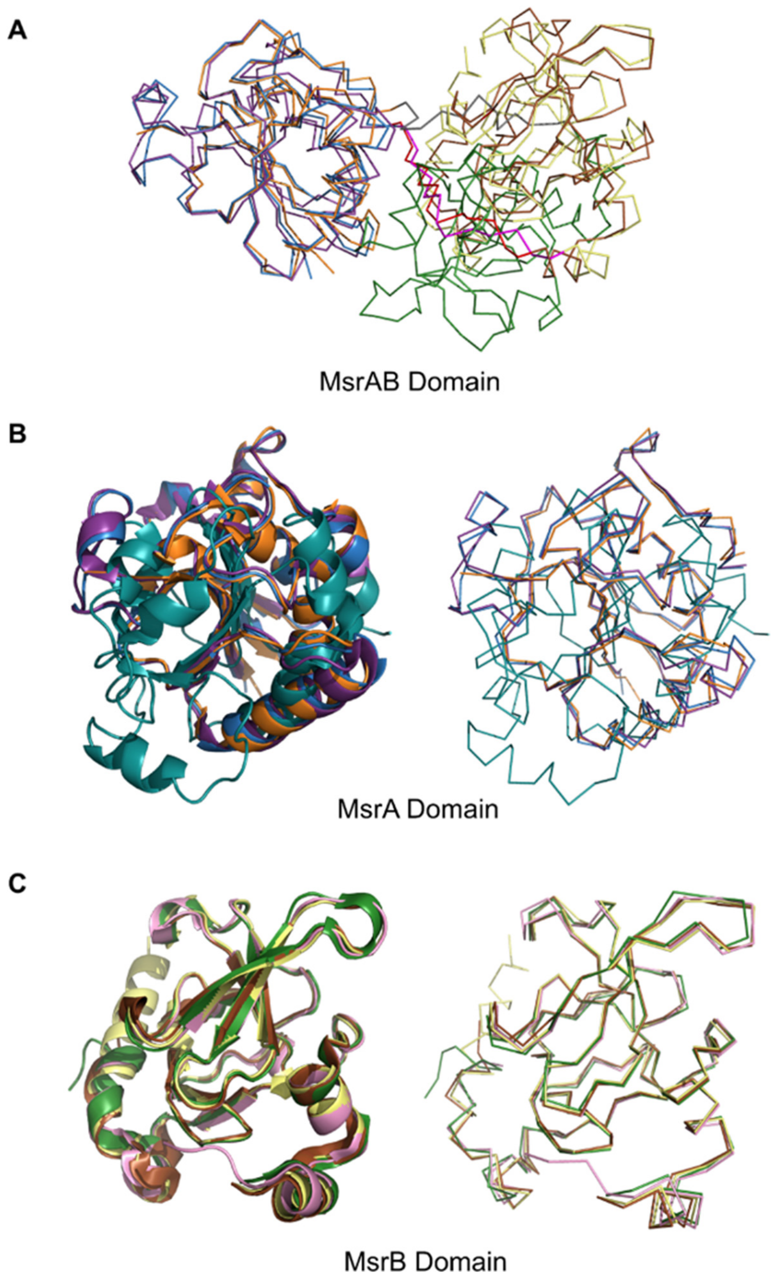

Figure S3: Comparison of *iloops* between HpMsrAB and SpMsrAB and superimposition of HpMsrAB and SpMsrAB by aligning the MsrA domains. Structural comparison of HpMsrAB with TdMsrAB and SpMsrAB. (**a**) Superimposition of HpMsrAB$^{C44S/C318S}$ with TdMsrAB$^{C11S/C285S}$ and SpMsrAB. It is represented as a ribbon model. The N-terminal MsrA domains (residues 34-192), TdMsrA (residues 1-159), and SpMsrA (residues 1-158)) are colored sky blue, orange, and violet-purple, respectively. The C-terminal MsrB domains (residues 206–357), TdMsrB (residues 173–317), and SpMsrB (residues 172–316)) are colored pale yellow, forest, and brown, respectively. The linker connecting HpMsrA and HpMsrB (residues 193-205), the linker connecting TdMsrA and TdMsrB (residues 160-172), and the linker connecting SpMsrA and SpMsrB (residues 159-171) are colored magenta, gray, and red, respectively. (**b**) Superimposition of HpMsrA^{C44S} with TdMsrA^{C11S} and SpMsrA. It is represented as illustration and ribbon models, respectively, and the colors are the same as in **(a).** (**c**) Superimposition of HpMsrB^{C318S} with TdMsrB^{C258S} and SpMsrB. It is represented as illustration and ribbon models, respectively, and the colors are the same as in **(a).**

Figure S4

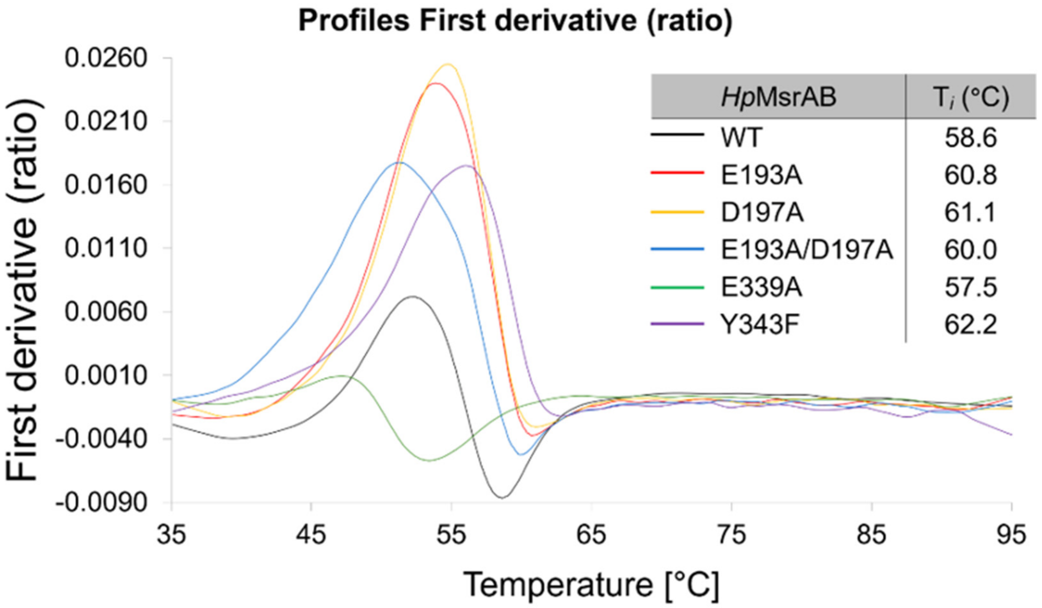

Figure S4: Inflection temperature (Ti) measurement. T_i of *Hp*MsrAB WT and mutants. The first derivatives of the fluorescence ratio (350 to 330 nm) were measured. Exact T_i values are listed in the table. Intrinsic tryptophan fluorescence of *Hp*MsrAB WT and mutants were monitored while increasing the temperature. Fluorescence ratio at 350/330 nm was calculated to determine the protein folding.

Figure S5

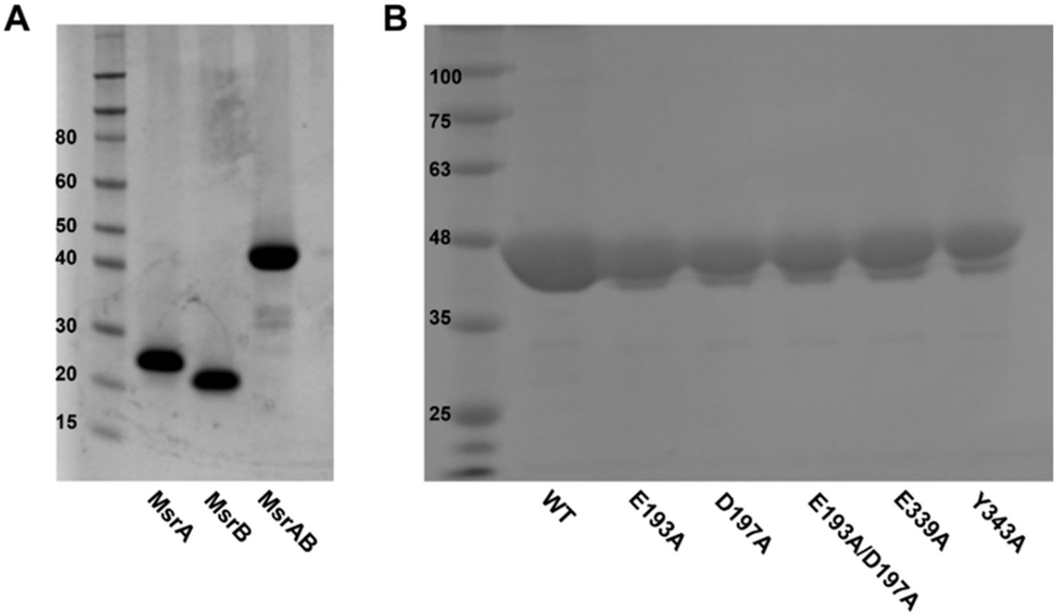

Figure S5: Image of SDS-gel electro-511 phoresis of WT and mutant HpMsrAB.
